# Neoadjuvant Systemic Therapy for Resectable Intrahepatic Cholangiocarcinoma: From Retrospective Studies to Randomized Evidence

**DOI:** 10.3390/cancers18172861

**Published:** 2026-09-04

**Authors:** Hironobu Suto, Asahiro Morishita, Hiroyuki Matsukawa, Mina Nagao, Takuro Fuke, Yoshio Shimizu, Yasuhisa Ando, Minoru Oshima, Ryosuke Imado, Mai Nakahara, Kyoko Oura, Tomoko Tadokoro, Koji Fujita, Joji Tani, Hideki Kobara, Kensuke Kumamoto, Keiichi Okano

**Affiliations:** 1 Department of Gastroenterological Surgery, Faculty of Medicine, Kagawa University, 1750-1 Ikenobe, Miki-cho, Kita-gun, Kagawa 761-0793, Japan; matsukawa.hiroyuki@kagawa-u.ac.jp (H.M.); nagao.mina.qu@kagawa-u.ac.jp (M.N.); fuke.takuro@kagawa-u.ac.jp (T.F.); shimizu.yoshio@kagawa-u.ac.jp (Y.S.); ando.yasuhisa.rf@kagawa-u.ac.jp (Y.A.); oshima.minoru@kagawa-u.ac.jp (M.O.); kumamoto.kensuke@kagawa-u.ac.jp (K.K.); okano.keiichi@kagawa-u.ac.jp (K.O.); 2 Department of Gastroenterology and Neurology, Faculty of Medicine, Kagawa University, 1750-1 Ikenobe, Miki-cho, Kita-gun, Kagawa 761-0793, Japan; morishita.asahiro@kagawa-u.ac.jp (A.M.); imado.ryosuke@kagawa-u.ac.jp (R.I.); nakahara.mai@kagawa-u.ac.jp (M.N.); oura.kyoko@kagawa-u.ac.jp (K.O.); tadokoro.tomoko@kagawa-u.ac.jp (T.T.); fujita.koji@kagawa-u.ac.jp (K.F.); tani.joji@kagawa-u.ac.jp (J.T.); kobara.hideki@kagawa-u.ac.jp (H.K.); 3 Department of Genome Medical Science and Medical Genetics, Faculty of Medicine, Kagawa University, 1750-1 Ikenobe, Miki-cho, Kita-gun, Kagawa 761-0793, Japan

**Keywords:** intrahepatic cholangiocarcinoma, neoadjuvant systemic therapy, perioperative therapy, liver resection, high-risk resectable disease, event-free survival, GOLP

## Abstract

Complete resection remains the only potentially curative treatment for localized intrahepatic cholangiocarcinoma (iCCA), yet postoperative recurrence is common, particularly in patients with high-risk disease. Neoadjuvant systemic therapy may permit earlier control of occult micrometastatic disease, optimize the delivery of systemic treatment, and provide an in vivo assessment of tumor biology prior to major hepatectomy. These potential benefits must be balanced against treatment-related toxicity, surgical delay, and the risk of disease progression precluding resection. Early evidence was primarily derived from retrospective studies, which yielded inconsistent survival outcomes and exhibited substantial vulnerability to confounding and treatment-selection bias. The single-arm NEO-GAP trial subsequently demonstrated the feasibility of administering neoadjuvant gemcitabine, cisplatin, and nab-paclitaxel followed by surgical resection. More recently, the randomized phase II–III ZSAB-neoGOLP trial showed that neoadjuvant gemcitabine–oxaliplatin, lenvatinib, and toripalimab followed by surgery prolonged median event-free survival compared with upfront surgery (median: 18.0 vs. 8.7 months) without substantially compromising surgical feasibility. However, the interim overall survival analysis was inconclusive, and the generalizability of these findings beyond selected, medically fit patients treated at Chinese centers remains uncertain. This narrative review critically appraises the evolving evidence, discusses patient selection and perioperative treatment, and identifies priorities for future research. Current evidence supports the selective consideration of neoadjuvant therapy in medically fit patients with technically resectable but oncologically high-risk iCCA, rather than its routine use in all resectable cases.

## Simple Summary

Intrahepatic cholangiocarcinoma is an aggressive bile duct cancer that arises within the liver and frequently recurs after surgery. Administering anticancer treatment before surgery may treat occult disease earlier and help identify patients most likely to benefit from major liver surgery; however, this approach risks causing treatment-related toxicity or permitting disease progression prior to resection. This review examines the evidence for this approach in patients whose tumors are surgically resectable but exhibit features associated with a high risk of recurrence. Early studies were retrospective and difficult to interpret because they inherently included highly selected patient cohorts. A recent randomized trial demonstrated that a preoperative multidrug regimen prolonged the time to progression, recurrence, or death, although a definitive overall survival benefit has not yet been established. These findings support the selective use of this approach in medically fit patients and highlight the need for extended follow-up, international validation, and refined patient-selection criteria.

## 1. Introduction

Intrahepatic cholangiocarcinoma (iCCA) is the second most common primary liver malignancy and is characterized by substantial clinical and biological heterogeneity [1]. Complete hepatic resection remains the only established treatment with the potential for a long-term cure for most patients with localized disease [1]. Nevertheless, recurrence after curative-intent surgery is common. In a multi-institutional cohort of 933 patients, 73.4% experienced disease recurrence, most within 2 years of resection [2]. In another international cohort, 22.3% experienced recurrence within 6 months, an outcome that was associated with particularly poor long-term survival [3]. More recently, only 16.4% of 635 patients who underwent resection were alive without recurrence beyond 5 years [4]. Large tumor size, multifocal or satellite disease, major vascular invasion, and regional lymph node metastasis have been consistently associated with early recurrence and failure to achieve durable disease control [1,2,3,4]. Thus, technical resectability does not necessarily imply favorable tumor biology or satisfactory outcomes with surgery alone.

Capecitabine has become a widely accepted postoperative systemic therapy for patients with resected biliary tract cancer based on the BILCAP trial and its long-term follow-up [5,6]. However, this evidence was generated in a heterogeneous population of patients with biliary tract cancer, and an exclusively postoperative strategy delays systemic treatment until recovery from major liver surgery. Neoadjuvant systemic therapy may enable earlier treatment of occult micrometastatic disease and in vivo assessment of tumor biology prior to major hepatectomy. These theoretical benefits must be balanced against treatment-related toxicity, delay of potentially curative surgery, and the risk that disease progression or clinical deterioration during therapy will preclude resection. Accordingly, the relevant clinical question is not simply whether preoperative therapy is biologically attractive, but whether the comprehensive neoadjuvant paradigm improves outcomes from the time treatment is initiated.

Initial evidence was derived mainly from retrospective registries and institutional cohorts. National Cancer Database analyses suggested an association between neoadjuvant treatment and improved overall survival in selected patients, particularly those with higher-risk disease [7,8]. However, these studies were limited by nonrandom treatment allocation, heterogeneous treatment regimens and eligibility criteria, incomplete characterization of baseline resectability, and frequent restriction of the analysis to patients who ultimately underwent resection. The multicenter, single-arm NEO-GAP trial subsequently demonstrated the feasibility of prospective neoadjuvant gemcitabine, cisplatin, and nab-paclitaxel in patients with high-risk resectable iCCA but could not establish comparative efficacy [9]. More recently, the randomized phase II–III ZSAB-neoGOLP trial demonstrated that neoadjuvant gemcitabine–oxaliplatin, lenvatinib, and toripalimab followed by surgery significantly prolonged event-free survival compared with upfront surgery, although the interim overall survival analysis remained statistically inconclusive [10].

Two previous meta-analyses suggested that neoadjuvant chemotherapy might improve selected survival outcomes; however, both were based entirely on nonrandomized studies, and the more recent analysis included both resectable and locally advanced disease [11,12]. Neither included the subsequently published randomized ZSAB-neoGOLP trial. The availability of randomized evidence therefore warrants a critical reappraisal of the earlier observational literature. This narrative review critically examines the rationale and evolution of the published evidence for neoadjuvant systemic therapy in resectable iCCA, with particular emphasis on how randomized data alter the interpretation of earlier observational findings, and it discusses clinical implications and key questions that should guide future investigation.

## 2. Rationale and Potential Risks of Neoadjuvant Systemic Therapy in Resectable iCCA

In this review, neoadjuvant therapy refers to systemic anticancer treatment administered prior to planned curative-intent resection in patients with iCCA considered technically resectable at baseline. This differs from conversion therapy, in which initially unresectable disease is treated to facilitate subsequent resection. The distinction is important because the target populations and appropriate outcome measures differ.

### 2.1. Potential Advantages

The principal rationale is the earlier treatment of occult disease. The high frequency of recurrence, including events occurring within the first 6–24 months after curative-intent resection, suggests that clinically undetectable dissemination is often present at the time of surgery [2,3,4]. Upfront resection removes the macroscopic tumor but postpones systemic treatment until postoperative recovery, whereas a neoadjuvant strategy begins systemic therapy immediately. This theoretical advantage is biologically plausible but requires validation through comparative clinical evidence.

A second potential advantage is the optimized delivery of systemic therapy. Postoperative trials in biliary tract cancer have yielded heterogeneous results [5,6,13,14,15], and an adjuvant-only strategy remains dependent on recovery from major surgery. This consideration is particularly relevant in iCCA, in which curative-intent resection often entails major hepatectomy and postoperative recovery may delay or preclude adjuvant treatment [1]. In the BILCAP trial, 55% of patients assigned to capecitabine completed all eight cycles [5]. In a National Cancer Database analysis, only 45% of patients who underwent resection for cholangiocarcinoma received adjuvant therapy, with a median interval of 59 days from surgery to treatment initiation [16]. Although these data are not iCCA-specific and do not prove the superiority of preoperative treatment, they illustrate the practical rationale for delivering at least a portion of the systemic therapy prior to hepatectomy.

Neoadjuvant therapy also permits an in vivo assessment of tumor biology. Disease control during a predefined treatment period may identify patients with treatment-sensitive tumors, whereas rapid progression or new metastatic disease may indicate that, despite technical resectability, major resection is unlikely to provide durable benefit. However, favorable survival among patients who ultimately undergo resection may partly reflect treatment-based biological selection rather than the direct effect of the regimen [17]. Preoperative treatment may therefore help avoid nonbeneficial surgery, but resection-only analyses can overestimate the benefit of the overall strategy.

Tumor shrinkage may facilitate resection in selected patients, but downstaging is not the principal objective when disease is already technically resectable. The similar microscopically margin-negative (R0) resection rates observed after neoadjuvant treatment and upfront surgery in the ZSAB-neoGOLP trial suggest that earlier systemic control and biological selection may be more important than improved margins [10].

### 2.2. Potential Risks and Methodological Implications

The principal concern is the loss of the opportunity for immediate curative-intent surgery due to disease progression, toxicity, or clinical deterioration. Accordingly, surgical attrition due to progression, toxicity, or clinical deterioration is an essential outcome measure of any neoadjuvant strategy [9,10].

Progression during treatment may reveal aggressive tumor biology that would have recurred rapidly after upfront surgery, but it may also preclude immediate resection in a patient who might otherwise have benefited. This counterfactual cannot be resolved by single-arm studies or analyses limited to patients who undergo resection. Outcomes should therefore be measured from randomization or treatment initiation and should include preoperative progression, toxicity, attrition prior to surgery, postoperative recurrence, and death. Event-free survival is particularly informative because it avoids the favorable selection bias inherent in recurrence-free survival calculated only among patients who undergo resection [10,17].

Before treatment initiation, histological confirmation, high-quality liver imaging, exclusion of extrahepatic disease, assessment of hepatic reserve and the future liver remnant, and multidisciplinary confirmation of technical resectability are essential [1]. Misclassification of initially unresectable disease would blur the distinction between neoadjuvant and conversion therapy, whereas treatment of low-risk resectable disease could expose patients to unnecessary toxicity and delay surgery without demonstrated benefit.

Overall, neoadjuvant systemic therapy may provide earlier systemic control and biological selection in technically resectable but oncologically high-risk iCCA. These potential benefits must be weighed against toxicity, surgical delay, and surgical attrition, and the entire treatment pathway should be evaluated from treatment initiation.

## 3. Evolution of Clinical Evidence

### 3.1. Retrospective Evidence

Prior to the availability of prospective trials, evidence for neoadjuvant therapy in resectable iCCA was derived from retrospective surgical cohorts and national databases. An early international, multi-institutional analysis included 1057 patients who underwent curative-intent hepatectomy, of whom only 62 (5.9%) received preoperative chemotherapy [18]. Although the neoadjuvant cohort presented with more advanced disease, postoperative morbidity and long-term outcomes were similar to those observed after upfront surgery. Propensity-matched analyses demonstrated numerically longer overall and disease-free survival after preoperative chemotherapy, but the differences were not statistically significant [18]. This study supported the feasibility of this approach but did not establish a survival advantage.

Subsequent National Cancer Database studies yielded mixed results. Utuama et al. reported no significant overall survival benefit in the overall propensity-matched cohort, although an association with improved survival emerged among patients with stage II–III disease [7]. Mason et al. reported lower mortality after neoadjuvant treatment among patients who ultimately underwent resection, whereas in the iCCA subgroup of a broader cholangiocarcinoma cohort, Parente et al. similarly observed improved adjusted survival compared with surgery alone, but no significant difference compared with surgery followed by adjuvant chemotherapy [8,19]. More recently, Wehrle et al. observed a greater lymph node yield and a lower pathological node-positivity rate after neoadjuvant systemic therapy, but no statistically significant overall survival advantage over upfront resection [20]. A complementary National Cancer Database analysis by Alaimo et al. examined staging concordance and downstaging among 3384 patients who underwent upfront surgery or neoadjuvant therapy followed by resection [21]. Of the 480 patients who received neoadjuvant therapy, an estimated 18.4% were downstaged. Median overall survival was longer with neoadjuvant therapy in patients with cT1–2N1 disease (31.5 vs. 22.4 months) and cT3–4N1 disease (27.8 vs. 14.4 months), while downstaging in cT3–4N1 disease was associated with a lower risk of death (HR 0.35). These findings further suggest that any survival signal may be concentrated in patients with clinically node-positive or more advanced disease; however, the analysis remained limited by its retrospective, resection-only design and reliance on registry-based staging. Because these studies utilized overlapping National Cancer Database eras, they should not be interpreted as independent replications. Their shared limitations included incomplete characterization of baseline resectability, treatment details, radiological response, and outcomes among patients who initiated therapy but did not undergo resection [7,8,19,20,21].

An institutional study provided more detailed clinical and pathological information but was limited by a small sample size. Sutton et al. reported an association between neoadjuvant chemotherapy and improved overall survival in 52 patients who underwent resection; however, only 10 received preoperative treatment, no significant recurrence-free survival benefit was observed, and major postoperative morbidity and positive resection margins were not increased [22]. This study supported surgical feasibility but failed to demonstrate a consistent comparative survival benefit.

An additional methodological limitation is that most retrospective studies were restricted to patients who ultimately underwent surgery. Dong et al. evaluated neoadjuvant therapy as a comprehensive treatment paradigm by including patients who initiated treatment but did not proceed to resection and by examining an external validation cohort [17]. Patients who completed therapy and underwent resection exhibited favorable overall survival, whereas those who experienced surgical attrition had outcomes comparable with those of patients treated palliatively. The authors interpreted this pattern principally as evidence of treatment-based biological selection rather than a direct treatment effect. The study therefore highlighted the dual role of neoadjuvant therapy as both treatment and selection, while demonstrating how resection-only analyses can overestimate clinical benefit [17].

Taken together, the retrospective literature supported the feasibility of systemic therapy prior to hepatectomy and suggested potential benefits in selected patients with high-risk disease [7,8,17,18,19,20,21,22]. Favorable survival associations were reported most consistently in patients with stage II–III or clinically node-positive disease [7,21], whereas findings in broader resected cohorts remained mixed [8,19,20]. However, survival results were not uniform, and the studies were vulnerable to confounding by indication, heterogeneous definitions of resectability and high-risk disease, variations in treatment regimens, and differences in the starting points used for survival analyses. Most importantly, the reliance on resection-only cohorts excluded patients who experienced preoperative progression, toxicity, or clinical deterioration. These design features introduced treatment-selection and potential immortal-time biases. The retrospective evidence was therefore hypothesis-generating: it provided a rationale for prospective evaluation but could not definitively determine whether neoadjuvant therapy was superior to upfront surgery. The principal retrospective studies are summarized in Table 1.

### 3.2. Prospective Feasibility Evidence: The NEO-GAP Trial

Prospective evaluation of this approach began with the NEO-GAP trial. The choice of the gemcitabine, cisplatin, and nab-paclitaxel (GAP) regimen was based on encouraging activity in a single-arm phase II study of advanced biliary tract cancer, which reported a partial response rate of 45% and a disease control rate of 84% [23]. NEO-GAP was a multi-institutional, single-arm phase II study that enrolled 30 evaluable patients with technically resectable, high-risk iCCA, defined by a tumor larger than 5 cm, multiple tumors, radiographic major vascular invasion, or regional lymph node involvement. Patients received four 21-day cycles of GAP prior to planned curative-intent resection, and the primary endpoint was the completion of both preoperative chemotherapy and surgery [9].

Twenty-two patients (73%) completed the planned neoadjuvant pathway. The radiological disease control rate was 90%, grade 3 or higher treatment-related adverse events occurred in 33% of patients, and 50% of patients required at least one dose reduction; no treatment-related deaths were reported [9]. These findings demonstrated the feasibility of the predefined neoadjuvant strategy. However, the eight patients who did not complete the full pathway underscored the need to evaluate neoadjuvant strategies from treatment initiation rather than only among patients who ultimately undergo resection.

NEO-GAP was designed to assess feasibility, not superiority over upfront surgery. Its small sample size and single-arm design precluded conclusions regarding comparative efficacy [9]. In the later randomized SWOG S1815 trial in advanced biliary tract cancer, GAP did not improve overall survival compared with gemcitabine and cisplatin and was associated with greater toxicity [24]. Although conducted in a different disease setting, this result cautions against interpreting the activity observed in NEO-GAP as evidence of regimen-specific superiority. The principal contribution of NEO-GAP was therefore to demonstrate the feasibility of a structured neoadjuvant strategy and to provide a prospective framework for the subsequent randomized ZSAB-neoGOLP trial [10].

### 3.3. Randomized Evidence: The ZSAB-neoGOLP Trial

The combination of gemcitabine, oxaliplatin, lenvatinib, and toripalimab (GOLP) was initially evaluated in a single-center, single-arm phase II study of 30 patients with advanced iCCA. The regimen achieved an objective response rate of 80%, although grade 3 or higher adverse events occurred in 56.7% of patients [25]. The randomized TOPAZ-1 and KEYNOTE-966 trials subsequently established the activity of chemoimmunotherapy in advanced biliary tract cancer [26,27], but neither validated GOLP itself nor its use in the perioperative setting.

The ZSAB-neoGOLP trial was an investigator-initiated, multicenter, open-label, randomized phase II–III trial conducted at 11 hospitals in China [10]. A total of 178 patients with technically resectable iCCA and at least one high-risk feature were randomly assigned to neoadjuvant GOLP followed by surgery or to upfront surgery. High-risk features included a tumor larger than 5 cm, radiographic vascular invasion, multiple intrahepatic tumors, suspected hilar lymph node metastasis, or elevated carbohydrate antigen 19-9 (CA19-9). Eligible patients were 18–70 years of age, had an Eastern Cooperative Oncology Group performance status score of 0, and had preserved hepatic function. The neoadjuvant group received three cycles of gemcitabine and oxaliplatin plus toripalimab and 9 weeks of lenvatinib prior to reassessment and planned resection. Eight cycles of postoperative capecitabine were planned in both groups. The trial therefore evaluated a complete perioperative strategy rather than the isolated effect of preoperative GOLP.

The primary endpoint was event-free survival measured from randomization, with events defined as progression precluding surgery, postoperative recurrence or metastasis, a second primary cancer, or death [10]. After a median follow-up of 16.9 months, median event-free survival was 18.0 months with neoadjuvant GOLP and 8.7 months with upfront surgery (*p* < 0.001). The estimated 24-month overall survival rates were 79% and 61%, respectively, with a hazard ratio for death of 0.43. However, the nominal *p* value of 0.005 did not cross the prespecified interim significance boundary of 0.0019; the overall survival benefit should therefore be regarded as encouraging but not yet definitive [10].

The regimen produced an objective radiological response in 55% of patients, with a major pathological response in 19% and a pathological complete response in 5% [10]. Surgery was performed in 97% of patients in the neoadjuvant group and 99% of those in the control group, and the R0 resection rates were 95% and 93%, respectively. Thus, the event-free survival advantage was unlikely to be explained by a substantial increase in either surgical completion or margin-negative resection rates. During the neoadjuvant phase, grade 3 or higher adverse events occurred in 28% of patients, and no treatment-related deaths were reported [10].

The ZSAB-neoGOLP trial provided the first randomized evidence that a neoadjuvant GOLP-based perioperative strategy improved event-free survival without significantly compromising surgical feasibility [10]. The evolution of prospective evidence from feasibility testing to randomized efficacy assessment is summarized in Table 2. The broader clinical applicability of this approach and the remaining uncertainties are considered below.

## 4. Clinical Implications and Remaining Questions

The available evidence supports the selective use of neoadjuvant systemic therapy in medically fit patients with technically resectable but oncologically high-risk iCCA, particularly those resembling the population enrolled in the ZSAB-neoGOLP trial [10]. It does not support its routine use for all patients with resectable disease.

### 4.1. Patient Selection and Treatment Pathway

Although no universally accepted definition of high-risk resectable disease has been established, the selection criteria used in the published prospective studies show substantial convergence. The NEO-GAP and ZSAB-neoGOLP both included a tumor larger than 5 cm, multifocal disease, vascular invasion, and regional lymph-node involvement, whereas ZSAB-neoGOLP additionally included elevated serum CA19-9 [9,10]. These shared features provide an emerging pragmatic framework for patient selection and may form the basis for future trial design and clinical recommendations. Nevertheless, these factors represent clinically distinct anatomical and biological conditions and may not predict the same magnitude of benefit from preoperative treatment.

Kawashima et al. proposed preoperative oncological resectability criteria based on tumor size, multinodularity, major vascular invasion, and radiographically suspected lymph node metastasis [28]. These criteria separated patients into groups with markedly different postoperative survival outcomes and may provide a useful framework for multidisciplinary risk assessment. However, these criteria were derived from patients undergoing upfront surgery and are prognostic rather than predictive; they do not establish which patients specifically benefit from neoadjuvant therapy [28]. This distinction has direct implications for trial design. Current high-risk definitions enrich recurrence risk but do not identify treatment-sensitive disease. A patient with a large solitary tumor may differ biologically from a patient with multifocal disease or suspected nodal metastasis, even when both satisfy the same binary high-risk definition. Future studies should report outcomes according to each qualifying feature and formally test treatment-by-subgroup interactions. Composite risk models may improve prognostic stratification, but a clinically useful selection tool must demonstrate predictive value—that is, the ability to identify patients who derive greater relative benefit from neoadjuvant therapy than from upfront surgery. Until such evidence is available, high-risk criteria should guide multidisciplinary discussion and trial eligibility rather than function as definitive treatment selection algorithms [9,10,28].

The patient profile most directly supported by current evidence is a medically fit patient presenting with histologically confirmed, technically resectable iCCA, no distant metastasis, and at least one high-risk clinical or radiological feature. Baseline resectability and the treatment plan should be confirmed by a multidisciplinary team [1]. Neoadjuvant therapy should also remain clearly distinguished from conversion therapy for initially unresectable disease.

GOLP is the only regimen supported by randomized evidence specifically in high-risk resectable iCCA [10]. NEO-GAP established the feasibility of preoperative GAP but did not demonstrate superiority over upfront surgery [9]. Because the randomized evidence is specific to GOLP, neither GAP nor other chemotherapy or chemoimmunotherapy regimens can be considered equivalent in the perioperative setting [10,23,24,25,26,27]. Conversely, the complexity of GOLP may limit implementation where individual agents are unavailable or intensive toxicity monitoring is impractical. Future trials should distinguish whether they are testing treatment timing, immune or targeted intensification, or both. Without such separation, the relative contributions of the preoperative setting and the multidrug regimen will remain conflated [10,23,24,25,26,27].

Both prospective trials used a short, predefined treatment course followed by reassessment [9,10]. After the planned treatment course, surgery may proceed provided that no new metastatic or technically unresectable disease has developed, the patient remains medically fit, and treatment-related toxicity has resolved. A partial radiological response need not be an absolute prerequisite for surgery; the maintenance of resectability and the absence of progression may be more clinically relevant. Conversely, progression or deterioration that precludes surgery must be counted as an outcome of the neoadjuvant strategy rather than excluded from the analysis [10,17].

### 4.2. Response Assessment and Postoperative Treatment

Pathological regression may provide useful information, but it is not validated as a surrogate for long-term survival or as a criterion for proceeding to surgery. Ayabe et al. observed a major pathological response after neoadjuvant chemotherapy, but it was not associated with recurrence-free or overall survival [29]. Thus, favorable pathological response findings should not yet be used in isolation to determine surgical eligibility or postoperative treatment.

Postoperative capecitabine was planned in both groups in the ZSAB-neoGOLP trial [10]. The trial should therefore be interpreted as evaluating a perioperative strategy comprising neoadjuvant GOLP, surgery, and postoperative capecitabine rather than the isolated effect of preoperative treatment. Randomized trials of postoperative systemic therapy have yielded mixed results [5,6,13,14,15], and none evaluated patients who had already received intensive neoadjuvant chemoimmunotherapy. The optimal postoperative regimen and duration after GOLP consequently remain undefined. From a clinical trial perspective, the complete perioperative sequence is the relevant unit of evaluation. Reporting preoperative response or postoperative outcomes in isolation does not capture cumulative toxicity, surgical recovery, or the proportion of patients who initiate and complete postoperative therapy. Future trials should report the completion of each treatment component, relative dose intensity, interval to surgery, postoperative morbidity, time to postoperative treatment, and reasons for discontinuation. Patient-reported outcomes and quality of life should also be included, because an event-free survival advantage accompanied by substantial toxicity or prolonged treatment burden may not provide the same net benefit across age and fitness groups. Such reporting would clarify whether preoperative intensification improves systemic treatment delivery across the entire course or merely shifts toxicity from the postoperative to the preoperative period [5,6,10,13,14,15,16].

Postoperative circulating tumor DNA (ctDNA) positivity has been associated with early recurrence in biliary tract cancer [30]. However, the available evidence includes heterogeneous primary tumor sites and relates mainly to postoperative surveillance; serial perioperative ctDNA assessment therefore remains investigational.

Dynamic biomarker assessment may be more informative than a single pretreatment value. In a multicenter cohort of patients who underwent resection, persistent postoperative CA19-9 elevation, rather than normalization after resection, was independently associated with shorter recurrence-free and overall survival [31]. This observation supports the concept that serial changes may reflect residual disease burden, but postoperative normalization cannot be extrapolated directly to tumor response during neoadjuvant therapy. Prospective perioperative studies should therefore measure CA19-9 levels at standardized time points and interpret changes alongside imaging, pathology, and ctDNA rather than as an isolated decision rule.

An integrated response framework may ultimately be more informative than any single marker. Radiological stability may coexist with meaningful pathological regression, whereas pathological response does not necessarily indicate eradication of molecular residual disease. Biomarker thresholds and treatment adaptations should be prespecified rather than derived post hoc in small cohorts. Until validated, these measures should complement, not replace, conventional assessments of resectability, medical fitness, and disease progression [10,29,30,31].

### 4.3. Perioperative Safety After Neoadjuvant Therapy

Available data provide preliminary reassurance regarding short-term surgical safety, although they remain limited. In an ACS-NSQIP propensity-matched analysis of 706 patients, Choi et al. found no significant increase in 30-day postoperative complications (60.1% vs. 55.3%; adjusted OR 1.24, 95% CI 0.87–1.76) or postoperative length of stay (8.56 vs. 9.27 days) after neoadjuvant chemotherapy [32]. A recent systematic review and meta-analysis by Risi et al. reported pooled postoperative mortality and severe morbidity rates of 1.2% and 19.1%, respectively, after preoperative systemic therapy [33]. In the neoadjuvant subgroup, the corresponding estimates were 3.1% and 9.0%, but each was based on only two studies and had wide confidence intervals; moreover, the overall analysis combined conversion and neoadjuvant settings.

These findings do not suggest a clear overall increase in short-term morbidity or mortality among selected patients who have had surgery, but liver-specific safety remains uncertain. A more recent ACS-NSQIP analysis by Jehan et al. found similar mortality and bile-leak rates after neoadjuvant chemotherapy and upfront surgery, but higher rates of post-hepatectomy liver failure after both major (19.1% vs. 10.3%) and minor hepatectomy (8.8% vs. 2.3%) [34]. The differing findings may reflect differences in study periods, endpoints, and analytic methods. Careful assessment of hepatic reserve, treatment-related liver injury, the future liver remnant, and the planned extent of hepatectomy therefore remains essential.

### 4.4. Generalizability and Research Priorities

Several factors limit the generalizability of the ZSAB-neoGOLP findings. Overall survival follow-up remains immature, and the trial enrolled selected patients aged 18–70 years with an Eastern Cooperative Oncology Group performance status score of 0 at experienced Chinese centers [10]. The feasibility and toxicity profile may differ in older patients, patients with impaired performance status or liver function, or patients undergoing complex hepatectomy.

The interpretation of event-free survival also requires longer follow-up. This endpoint appropriately captures progression that precludes surgery as well as postoperative recurrence and death, but mature overall survival data are necessary to determine whether earlier disease control translates into durable benefit. Future trials should additionally report recurrence patterns, subsequent therapies, and the completion of the full perioperative strategy. Randomization or treatment initiation should remain the time origin for all principal analyses to avoid the selection biases that affected earlier retrospective studies [10,17].

Finally, future development should move beyond simply reproducing the same four-drug regimen. Molecular profiling has shown that iCCA comprises biologically diverse subgroups with potentially actionable alterations, including IDH1 mutations and FGFR2 rearrangements [35]. However, the predictive relevance of these alterations for neoadjuvant chemotherapy, immunotherapy, or antiangiogenic treatment is unknown. Randomized perioperative studies should therefore incorporate prospective tissue and blood collection, evaluate simpler and more globally available regimens, and prespecify translational analyses of treatment response and residual disease. The objective should be to identify the least burdensome yet effective strategy for the patients most likely to benefit, rather than to increase neoadjuvant treatment indiscriminately.

## 5. Conclusions

Neoadjuvant systemic therapy for resectable intrahepatic cholangiocarcinoma has progressed from retrospective evidence limited by selection bias to prospective feasibility testing and randomized evaluation. NEO-GAP demonstrated the feasibility of a predefined neoadjuvant strategy, whereas the ZSAB-neoGOLP trial provided the first randomized evidence that a GOLP-based perioperative strategy improves event-free survival without significantly compromising surgical feasibility. However, the current evidence does not support the routine use of neoadjuvant treatment for all patients with resectable iCCA. A definitive overall survival benefit has not yet been established, and the generalizability of GOLP beyond the selected, medically fit Chinese trial population remains uncertain. The optimal definition of high-risk disease, the most appropriate regimen, and the postoperative treatment strategy remain unresolved.

At present, neoadjuvant systemic therapy should be considered selectively for medically fit patients with technically resectable but oncologically high-risk iCCA following a multidisciplinary review. Mature survival data, external validation, and more precise patient selection criteria are required prior to broader implementation.

## Figures and Tables

**Table 1:** Retrospective Studies of Neoadjuvant Therapy for Resectable or High-Risk Intrahepatic Cholangiocarcinoma.

Study and Design	Treatment and Comparator	Main Findings	Key Limitation
A. Resection-based comparative cohorts			
Buettner et al., 2017 [18]. International multicenter cohort; 1057 resected patients; 62 (5.9%) received preoperative chemotherapy.	Preoperative chemotherapy vs. no preoperative chemotherapy.	Perioperative outcomes were similar; matched OS and DFS were numerically longer but not statistically significant.	Resection-only cohort; small treated group; heterogeneous regimens; residual selection bias.
Utuama et al., 2021 [7]. NCDB cohort; 881 resected patients; 73 (8.3%) received neoadjuvant chemotherapy.	Neoadjuvant chemotherapy vs. upfront surgery.	No significant OS benefit overall; longer OS was associated with treatment in stage II–III disease (HR 0.58).	Subgroup finding; resection-only cohort; limited treatment and baseline resectability details.
Mason et al., 2021 [8]. NCDB cohort; 4456 surgically resected patients.	Chemotherapy alone or chemoradiotherapy before surgery vs. upfront surgery.	Lower mortality after propensity matching (HR 0.77) and inverse probability weighting (HR 0.83).	Mixed modalities; resection-only cohort; incomplete regimen, response, and attrition data; overlap with other NCDB analyses.
Sutton et al., 2021 [22]. Single-center cohort; 52 resected patients; 10 received neoadjuvant chemotherapy.	Mainly gemcitabine–cisplatin vs. upfront surgery.	Improved OS (adjusted HR 0.16) but not RFS; major morbidity and positive margins were not increased.	Very small treated cohort; nonrandom allocation; resection-only analysis; residual confounding.
Parente et al., 2023 [19]. NCDB cohort; iCCA subgroup included 3772 patients, of whom 399 received neoadjuvant chemotherapy.	Neoadjuvant chemotherapy plus surgery vs. surgery alone or surgery plus adjuvant chemotherapy.	Adjusted OS was better than with surgery alone (HR 0.75) but not significantly different from surgery plus adjuvant chemotherapy (HR 1.19; *p* = 0.068).	Mixed primary sites; registry-defined treatments; resection-only cohort; limited regimen and response data.
Wehrle et al., 2024 [20]. NCDB propensity-matched cohort of resected stage I-IIIB iCCA with lymphadenectomy.	Neoadjuvant systemic therapy vs. upfront resection.	Greater lymph-node yield and lower node positivity, but no significant OS advantage.	Registry study; resection-only cohort; limited treatment details; overlapping NCDB era.
Alaimo et al., 2023 [21]. NCDB Bayesian analysis; 3384 resected patients; 480 (14.2%) received neoadjuvant therapy.	Neoadjuvant therapy followed by resection vs. upfront surgery; staging concordance and downstaging were assessed.	An estimated 18.4% were downstaged. Median OS was longer with neoadjuvant therapy in cT1–2N1 and cT3–4N1 disease; downstaging in cT3–4N1 disease was associated with lower mortality (HR 0.35).	Retrospective resection-only registry analysis; Bayesian estimation of downstaging; limited treatment and surgical-attrition data; overlap with other NCDB eras.
B. Treatment-initiation analysis including failure to reach resection			
Dong et al., 2026 [17]. Mayo Clinic cohort (425 patients) with MD Anderson validation (203 patients); 172 high-risk patients initiated neoadjuvant therapy.	Chemotherapy- or chemoimmunotherapy-based treatment; comparisons with high-risk upfront surgery and palliative treatment.	Among 172 patients initiating therapy, 96 underwent resection and 76 (44.2%) did not. Resected patients had longer median OS than high-risk upfront-surgery patients (68.6 vs. 34.9 months), whereas RFS was similar; nonresected patients had outcomes comparable to palliative treatment.	Nonrandomized and regimen-heterogeneous; favorable outcomes among resected patients may largely reflect treatment-based biological selection.

Studies are grouped according to the denominator used for outcome assessment. Resection-based cohorts included only patients who ultimately underwent surgery, whereas the treatment-initiation cohort included patients who began neoadjuvant therapy regardless of whether resection was achieved. NCDB analyses used partially overlapping study periods and should not be interpreted as independent validation cohorts. Abbreviations: DFS, disease-free survival; HR, hazard ratio; iCCA, intrahepatic cholangiocarcinoma; NCDB, National Cancer Database; OS, overall survival; RFS, recurrence-free survival.

**Table 2:** Evolution of Prospective Evidence for Neoadjuvant Systemic Therapy in High-Risk Resectable Intrahepatic Cholangiocarcinoma.

Study and Evidence Stage	Design, Treatment, and Primary Endpoint	Key Findings	Interpretation and Unresolved Issues
Maithel et al., 2023; NEO-GAP [9] Prospective feasibility	Multicenter, single-arm phase II study; 30 evaluable patients. Four 21-day cycles of gemcitabine, cisplatin, and nab-paclitaxel followed by planned resection. Primary endpoint: completion of both chemotherapy and surgery.	Twenty-two patients (73%) completed the pathway; disease-control rate 90%; grade ≥3 treatment-related adverse events 33%; no treatment-related deaths.	Demonstrated feasibility of a predefined chemotherapy-to-surgery pathway. The noncomparative design did not establish superiority over upfront surgery; 27% did not complete the full pathway.
Shi et al., 2026; ZSAB-neoGOLP [10] Randomized comparative efficacy	Multicenter randomized phase II–III trial; 178 patients. Three cycles of GEMOX plus toripalimab and 9 weeks of lenvatinib followed by surgery vs. upfront surgery; postoperative capecitabine planned in both groups. Primary endpoint: EFS from randomization.	Median EFS 18.0 vs. 8.7 months (*p* < 0.001); surgery 97% vs. 99%; grade ≥3 adverse events 28% during neoadjuvant treatment (treatment-related, 26%); no treatment-related deaths. Interim OS favored GOLP but did not meet the prespecified significance boundary.	First randomized evidence of an EFS benefit without substantial surgical attrition. Definitive OS benefit, external generalizability, contribution of each regimen component, and optimal postoperative strategy remain uncertain.

These trials addressed different clinical questions and are presented to illustrate the progression from feasibility testing to randomized efficacy assessment. The table should not be interpreted as a direct comparison of GAP and GOLP. Abbreviations: EFS, event-free survival; GAP, gemcitabine, cisplatin, and nab-paclitaxel; GEMOX, gemcitabine plus oxaliplatin; GOLP, gemcitabine–oxaliplatin, lenvatinib, and toripalimab; OS, overall survival.

## Data Availability

No new data were created or analyzed in this study. Data sharing is not applicable to this article.

## Abbreviations

The following abbreviations are used in this manuscript:

BILCAP	Biliary Tract Cancer Trial
CA19-9	Carbohydrate antigen 19-9
ctDNA	Circulating tumor DNA
ECOG	Eastern Cooperative Oncology Group
GAP	gemcitabine, cisplatin, and nab-paclitaxel
GOLP	Gemcitabine, oxaliplatin, lenvatinib, and toripalimab
iCCA	Intrahepatic cholangiocarcinoma
NEO-GAP	Neoadjuvant Gemcitabine, Cisplatin, and Nab-Paclitaxel Trial
R0	Microscopically margin-negative (resection)
SWOG	Southwest Oncology Group
ZSAB-neoGOLP	Zhongshan Hospital Neoadjuvant GOLP Trial
